# Luminescent Ruthenium(II) Complex Bearing Bipyridine and N-Heterocyclic Carbene-based C^∧^N^∧^C Pincer Ligand for Live-Cell Imaging of Endocytosis

**DOI:** 10.1038/srep09070

**Published:** 2015-03-13

**Authors:** Wai-Kuen Tsui, Lai-Hon Chung, Matthew Man-Kin Wong, Wai-Him Tsang, Hoi-Shing Lo, Yaxiang Liu, Chung-Hang Leung, Dik-Lung Ma, Sung-Kay Chiu, Chun-Yuen Wong

**Affiliations:** 1 Department of Biology and Chemistry, City University of Hong Kong, Tat Chee Avenue, Kowloon, Hong Kong SAR; 2 Department of Biomedical Sciences, City University of Hong Kong, Tat Chee Avenue, Kowloon, Hong Kong SAR; 3 State Key Laboratory of Quality Research in Chinese Medicine, Institute of Chinese Medical Sciences, University of Macau, Macao, China; 4 Department of Chemistry, Hong Kong Baptist University, 224 Waterloo Road, Kowloon, Hong Kong SAR

## Abstract

Luminescent ruthenium(II)-cyanide complex with N-heterocyclic carbene pincer ligand C^∧^N^∧^C = 2,6-bis(1-butylimidazol-2-ylidene)pyridine and 2,2′-bipyridine (bpy) shows minimal cytotoxicity to both human breast carcinoma cell (MCF-7) and human retinal pigmented epithelium cell (RPE) in a wide range of concentration (0.1–500 μM), and can be used for the luminescent imaging of endocytosis of the complex in these cells.

Endocytosis, a process that materials are internalized into cells without passing through the cell membranes, represents one of the most common mechanisms for cells to interact with their environments^1^. This mechanism is known in many vital cellular processes including nutrient acquisition, receptor desensitization, and antigen presentation^2^. Visualization of endocytosis would not only allow investigation of many physiological processes, but may also provide insight on the rational design of new therapeutic agents^3^,^4^. In this regard, designing luminescent molecules and visualizing how they are trafficked in cells upon they have been endocytosed using microscopic techniques would be a powerful way to understand endocytosis.

In these two decades, a new class of structurally robust and easily accessible complexes has been intensively studied on their potentials in biomedical applications. A reservoir of successful cases has been documented in dozens of reviews^5^,^6^,^7^,^8^,^9^,^10^,^11^. Yet, only a few examples have been reported with Ru(II)-NHC complexes and these studies are mainly focused on cytotoxicity evaluation^12^,^13^,^14^,^15^,^16^,^17^,^18^,^19^,^20^,^21^. In this regard, development of luminescent Ru(II)-NHC compounds for probing cellular processes may provide some insights for biomedical practice of coordination compounds.

In these years, our group have initiated a program to develop new luminescent organometallic Ru(II)- and Os(II)-bipyridine complexes^22^,^23^,^24^,^25^,^26^,^27^,^28^,^29^,^30^,^31^,^32^. Recently, we have developed luminescent Ru(II)-bipyridine complexes bearing neutral tridentate NHC-based pincer ligands 2,6-bis(1-butylimidazol-2-ylidene)pyridine (C^∧^N^∧^C)^30^. We envision that Ru(II) complexes in the form of [Ru^II^(C^∧^N^∧^C)(bpy)L]^n+^ would be a valuable system for the design of luminescent molecular probes for live-cell imaging, as the C^∧^N^∧^C and bpy ligands on the [Ru^II^(C^∧^N^∧^C)(bpy)L]^n+^ are inert towards ligand substitution, therefore would in principle minimize the chance of complicating any biological pathways via ligand dissociation. However, the [Ru^II^(C^∧^N^∧^C)(bpy)L]^n+^ complexes we reported previously (L = Cl^−^, n = 1; L = CH_3_CN, *t*-BuNC, n = 2) suffer from low luminescent quantum yields (10^−5^–10^−3^), and the ligand L is substitutionally labile in the case of L = CH_3_CN, making them imperfect molecular systems for live-cell imaging probes. We herein report the synthesis of [Ru^II^(C^∧^N^∧^C)(bpy)(C≡N)]^+^ (**1**), a complex which has improved quantum yield and non-labile auxiliary ligand (L = ^−^CN). It is not only non-toxic for both human breast carcinoma cell (MCF-7) and immortalized noncancerous human retinal pigmented epithelium cell (RPE), but can also be used for the luminescent imaging of endocytosis of the complex in these cells.

The design of Ru(II) complex [Ru^II^(C^∧^N^∧^C)(bpy)(C≡N)]^+^ (**1**) is a rational modification of our previously reported [Ru^II^(C^∧^N^∧^C)(bpy)L]^n+^ system (L = Cl^−^, n = 1; L = CH_3_CN, *t*-BuNC, n = 2). For complexes bearing [Ru^II^(bpy)] moiety, it is well-known that the presence of thermally-populated metal-centered ^3^dd excited state serve as an efficient deactivation pathway for the emissive triplet Ru-to-bpy metal-to-ligand charge transfer excited state (^3^MLCT)^33^,^34^,^35^,^36^,^37^,^38^,^39^,^40^. One promising approach to improve the population of the ^3^MLCT state is the incorporation of a stronger donor to elevate the ^3^dd state, and we therefore employ cyanide as L in this work.

## Results

### Synthesis

**Figure 1:**
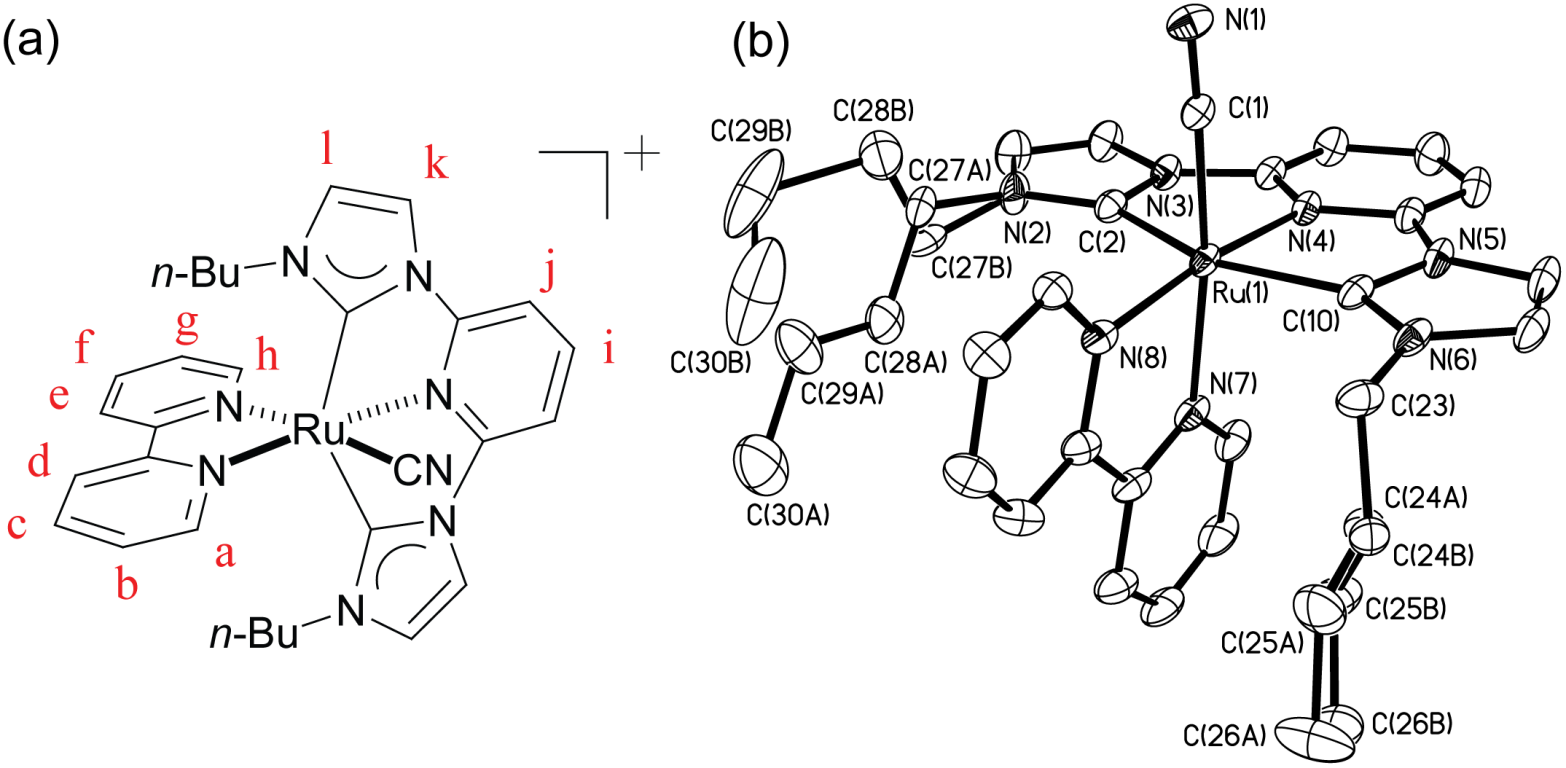
(a) Chemical structure and labeling scheme for the H and C atoms in complex **1**. (b) Perspective view of complex **1** at 30% probability ellipsoids; hydrogen atoms are omitted for clarity. The butyl chains on the Ĉ[Ncirc]C ligand are disordered over two positions (labeled as A and B, occupancy ratio ~ 0.7:0.3). Selected bond lengths (Å) and angles (deg): Ru(1)–C(1) 2.002(3), C(1)–N(1) 1.160(3), Ru(1)–C(2) 2.054(3), Ru(1)–C(10) 2.063(3), Ru(1)–N(4) 2.014(2), C(2)–Ru(1)–N(4) 77.22(10), C(10)–Ru(1)–N(4) 77.35(10).

Complex **1** was synthesized by reacting [Ru^II^(C^∧^N^∧^C)(bpy)(OH_2_)]^2+^ with KCN in refluxing H_2_O. Its ^1^H and ^13^C signals signify the presence of a plane of symmetry on NMR time scale at room temperature (e.g. 16 sets of aromatic signals in ^13^C spectrum). The ^13^C NMR signal at 194.7 ppm is typical for metalated N-heterocyclic carbene on Ru(II) complexes. Molecular structure of **1** was determined by X-ray crystallography and the perspective view is depicted in Figure 1. The Ru-center adopts a distorted octahedral geometry with C^∧^N^∧^C-pincer chelating meridionally in an almost planar configuration. The bond distances of Ru–C_NHC_ and Ru–C_CN_ are 2.054(3)–2.063(3) and 2.002(3) Å respectively. These Ru–C_NHC_ distances are similar to those in chloride-ligated complexes [Ru^II^(C^∧^N^∧^C)(N^∧^N)Cl]^+^ (N^∧^N = 2,2′-bipyridine-like diimine ligands, Ru–C_NHC_ = 2.048(2)–2.062(4) Å)^30^.

### Photophysical properties

**Figure 2:**
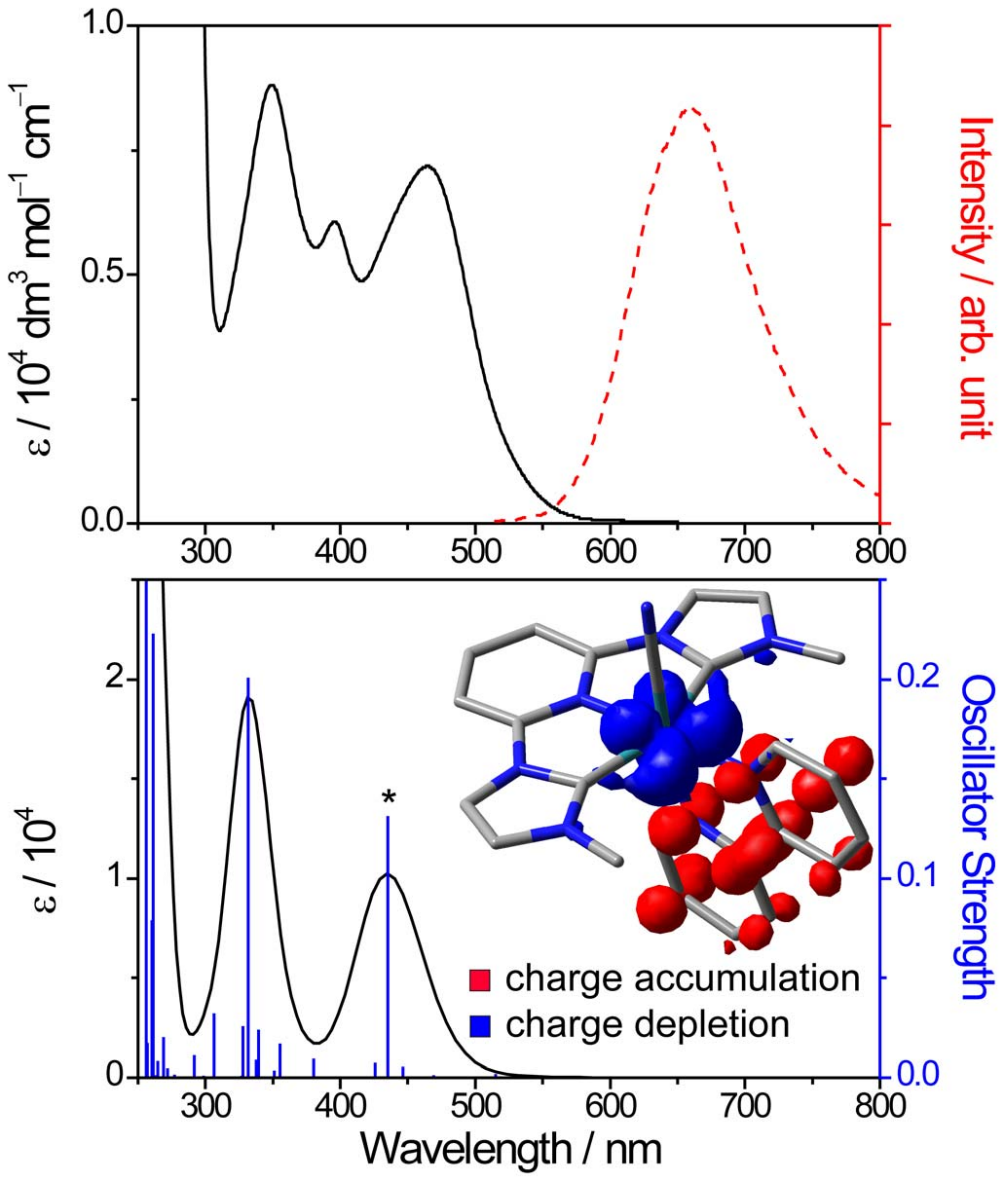
(a) Absorption (solid line) and emission (dash line, λ_ex_ = 450 nm) spectra of **1** in degassed CH_3_CN at 298 K. (b) TD-DFT calculated absorption spectrum for model complex **1′** in CH_3_CN. Excitation energies and oscillator strengths are shown by the blue vertical lines; spectrum (in black) is convoluted with a Gaussian function having a full width at half-maximum of 3000 cm^−1^. Insert shows the electronic difference density plot for **1′** at the vertical transition marked with * (isodensity value = 0.004 au).

The absorption and emission spectra for **1** are depicted in Figure 2a. With reference to our previous spectroscopic studies on the [Ru^II^(C^∧^N^∧^C)(bpy)L]^n+^ system, the lowest-energy absorption band (λ_max_ = 464 nm, ε_max_ = 7.1 × 10^3^ dm^3^ mol^−1^ cm^−1^) is assigned as d_π_(Ru^II^) → π*(bpy) MLCT transition^30^. The assignment is further supported by time-dependent density functional theory (TD-DFT) calculation on model complex **1′**, where its metal core is the same as that in **1** except the butyl chains on the C^∧^N^∧^C are replaced by methyl groups to reduce the computational cost. The simulated spectrum (Figure 2b) is in qualitative agreement with its experimental spectrum. The lowest energy transition for **1′** is a net d_π_(Ru^II^) → π*(bpy) charge transfer, which is clearly shown by an electronic difference density plot for **1′** (generated by taking the difference in the excited-state electron density and ground-state electron density) in its lowest energy excited state (marked with * in Figure 2). Complex **1** is emissive with λ_em_ at 659 nm in CH_3_CN, and 648 nm in CH_3_CN-H_2_O 1:1 v/v mixture, upon photoexcitation at its lowest-energy absorption band. Importantly, its emission quantum yield (8.23 × 10^−3^ in degassed CH_3_CN, 2.14 × 10^−3^ in non-degassed CH_3_CN-H_2_O mixture) and lifetime (1.71 μs in degassed CH_3_CN, 0.34 μs in non-degassed CH_3_CN-H_2_O mixture) are more superior to its Cl^−^, CH_3_CN^−^, and *t*-BuNC-ligated analogues (≤2.98 × 10^−3^ and ≤0.32 μs respectively in degassed CH_3_CN)^31^.

### Bio-imaging study

**Figure 3:**
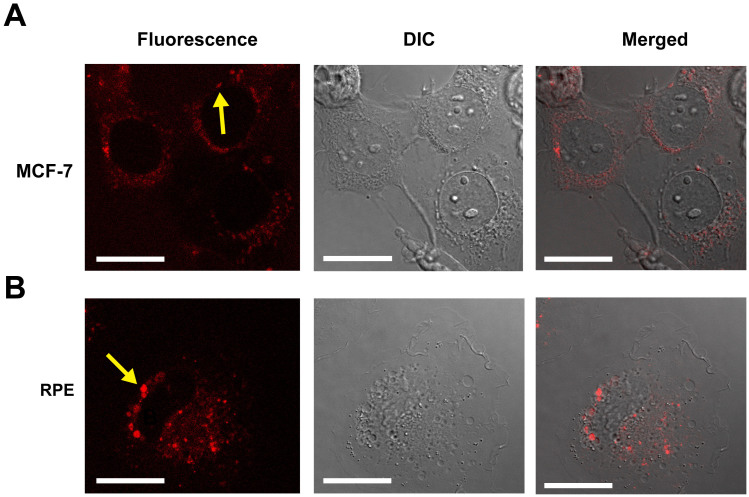
Luminescent images, differential interference contrast (DIC) images, and the overlay of these images (merged) of the live MCF-7 cells (A) and RPE cells (B) cells incubated with 1. Two examples of the mapping of luminescent signals to vesicles present in the cytoplasm are indicated by arrows. Scale bars represent 25 μm.

The interactions of complex **1** with mammalian cells were investigated by using the human mammary carcinoma cell line, MCF-7, and immortalized noncancerous human retinal pigmented epithelium (RPE) cell line. The toxicity of the complex to these cell lines was examined by employing the Prestoblue reagent, and the results showed that the complex was not cytotoxic to both cell lines in the concentration range of 0.1–500 μM (Figures S1 and S2). The luminescent bioimaging potential of **1** was evaluated using MCF-7 cells and RPE cells, the latter of which have a much flatter morphology than MCF-7 cells and thus allow easier observation of the transport of the complex in the cytoplasm of the cells. The localization of the luminescent signals of the complex in RPE cells was studied at complex concentration of 50, 100, 250, and 500 μM for 4 h, and surprisingly, the intensities of the luminescent signals presented in the cytoplasm of the cells were quite similar. Most of the signal appeared as spots under the laser confocal microscope and they could be mapped within the vesicles presented in the cytoplasm of the treated cells (Figure 3, lower row). The same observation was also obtained when the complex was incubated with MCF-7 cells (Figure 3, upper row). We also observed that complex **1** in the form of vesicles seemed to be more concentrated closer to the Golgi apparatus and nuclei with time of incubation. To confirm this observation, time-lapse luminescent micrographs were taken over an hour on the complex-treated RPE cells. The luminescent signals in the form of vesicles were observed to translocate from the distal end of the cell towards the nucleus (Figure S3), suggesting that the complex was engulfed from the medium (too weak to be recorded under confocal microscope), entered the cells via endocytosis in the form of endocytic vesicles, and transported along the microtubules towards the Golgi area, where the complex may then be processed or broken down in the lysosomes.

## Discussion

The results of this study show that the luminescent complex [Ru^II^(C^∧^N^∧^C)(bpy)(C≡N)]^+^ can be used as a bioimaging tool to illuminate the pathway that how molecules present in the surrounding enter the cells via the formation of endocytic vesicles and be processed along the endomembrane systems in the cells. Progress will be made to search for molecules that can change their emission wavelength by a change in pH, which would be very useful in showing when the vesicles merge with the acidic content of the lysosomes during the later stage of the endocytic pathway.

## Methods

### General Procedure

All reactions were performed under an argon atmosphere using standard Schlenk techniques unless otherwise stated. All reagents and solvents were used as received. Complex [Ru(C^∧^N^∧^C)(bpy)(OH_2_)](ClO_4_)_2_ was prepared according to literature method^30^. ^1^H, ^13^C{^1^H}, DEPT-135, ^1^H–^1^H COSY, and ^1^H–^13^C HSQC NMR spectra were recorded on Bruker 400 DRX FT-NMR spectrometer. Peak positions were calibrated with solvent residue peaks as internal standard. Electrospray mass spectrometry was performed on a PE-SCIEX API 3000 triple quadrupole mass spectrometer. Infrared spectra were recorded as KBr plates on an Avatar 360 FTIR spectrometer. UV–visible spectra were recorded on a Shimadzu UV-1700 spectrophotometer. Elemental analyses were done on an Elementar Vario Micro Cube carbon–hydrogen–nitrogen elemental micro-analyzer. Steady-state emission spectra were obtained on a Jobin Yvon Fluorolog-3-TCSPC spectrophotometer. Sample and standard solutions were degassed with at least three freeze-pump-thaw cycles. The emission quantum yields were measured by the method of Demas and Crosby^41^ with [Ru(bpy)_3_](PF_6_)_2_ in degassed CH_3_CN as standard (Φ_r_ = 0.062) and calculated by Φ_s_ = Φ_r_(B_r_/B_s_)(n_s_/n_r_)^2^(D_s_/D_r_), where the subscripts s and r refer to sample and reference standard solution, respectively, n is the refractive index of the solvents, D is the integrated intensity, and Φ is the luminescence quantum yield. The quantity B is calculated by B = 1 − 10^−AL^, where A is the absorbance at the excitation wavelength and L is the optical path length.

### Synthesis

**[Ru(C^∧^N^∧^C)(bpy)(CN)](ClO_4_), 1(ClO_4_).** A mixture of [Ru(C^∧^N^∧^C)(bpy)(OH_2_)](ClO_4_)_2_ (0.125 mmol) and KCN (0.25 mmol) was refluxed in water (30 mL) under argon for 2 h. Upon cooling to room temperature, a saturated NaClO_4_ solution (5 mL) was added into the reaction mixture to give bright orange solids. The solid was filtered and washed with EtOH/Et_2_O mixture (1:10, v/v) (2 × 5 mL) and Et_2_O (2 × 5 mL), and was recrystallized by slow diffusion of Et_2_O into CH_3_CN solution to give bright orange crystal. Yield 81 mg, 92%. Anal. Calcd for C_30_H_33_N_8_RuClO_4_: C, 51.03; H, 4.71; N, 15.87. Found: C, 50.97; H, 5.00; N, 15.92. ^1^H NMR (400 MHz, CD_3_CN): δ 0.57–0.86 (m, 10H, Bu), 0.88–1.04 (m, 2H, Bu), 1.25–1.43 (m, 2H, Bu), 3.20–3.37 (m, 4H, Bu), 7.04–7.09 (m, 1H, H_g_), 7.13 (d, *J* = 2.4 Hz, 2H, H_k_/H_l_), 7.23–7.28 (m, 1H, H_h_), 7.62–7.70 (m, 3H, H_b_ + H_j_), 7.77–7.84 (m, 1H, H_f_), 7.94 (d, *J* = 2.4 Hz, 2H, H_k_/H_l_), 7.99–8.10 (m, 2H, H_c_ + H_i_), 8.29–8.35 (m, 1H, H_e_), 8.41–8.47 (m, 1H, H_d_), 10.10–10.17 (m, 1H, H_a_). ^13^C NMR (100.6 MHz, CD_3_CN): δ 13.75, 20.23, 34.31, 50.78 (Bu), 106.71 (C_j_), 118.09 (C_k_/C_l_), 123.59 (C_e_), 123.90 (C_k_/C_l_), 124.44 (C_d_), 126.51 (C_g_), 127.12 (C_b_), 135.65 (C_c_), 136.89 (C_f_), 139.51 (C_i_), 143.66 (C≡N), 150.07 (C_h_), 154.47 (quaternary carbon), 155.05 (C_a_), 155.84, 156.33 (quaternary carbons), 194.73 (Ru–*C*_NHC_). IR (KBr, cm^−1^): *ν*_CN_ = 2063. ESI-MS: *m*/*z* 607.5 [M^+^].

### X-ray Crystallography

X-ray diffraction data for **1**(ClO_4_) was collected on an Oxford Diffraction Gemini S Ultra X-ray single crystal diffractometer with Mo K*α* radiation (λ = 0.71073 Å) at 173 K. The data was processed using CrysAlis. The structure was solved by Patterson method, and refined by full-matrix least-squares based on *F*^2^ with program SHELXS-97 and SHELXL-97 within WinGX. All non-hydrogen atoms were refined anisotropically in the final stage of least-squares refinement. The positions of H atoms were calculated based on riding mode with thermal parameters equal to 1.2 times that of the associated C atoms. The butyl chains on the C^∧^N^∧^C ligand are disordered over two positions, and split model was applied. CCDC 1043253 contains the supplementary crystallographic data for this paper, which can be obtained free of charge from The Cambridge Crystallographic Data Centre via www.ccdc.cam.ac.uk/data_request/cif.

### Computational Methodology

DFT and TD-DFT calculations were performed on model complex **1′** using the ORCA software package (version 3.0.0). Its electronic ground state was optimized using the PBE0 functional^42^,^43^ accompanied with (i) the zero-order regular approximation (ZORA)^44^,^45^,^46^ to account for relativistic effects, (ii) the conductor-like screening model (COSMO)^47^ to model solvation in CH_3_CN, and (iii) atom-pairwise dispersion correction with Becke-Johnson damping^48^,^49^. The def2-SVP basis sets were used for the H, C, and N atoms, while the def2-TZVP(-f) basis set was used for the Ru atom^50^. Auxiliary basis sets, used to expand the electron density in the calculations, were chosen to match the orbital basis sets^51^,^52^. The combination of the resolution of the identity and the “chain of spheres exchange” algorithms (RIJCOSX)^53^,^54^,^55^ was used to accelerate all DFT and TD-DFT calculations. Tight SCF convergence criteria (1 × 10^−8^ *E*_h_ in energy, 1 × 10^−7^ *E*_h_ in the density charge, and 1 × 10^−7^ in the maximum element of the DIIS error vector) were used throughout. The vertical transition energies for **1′** in CH_3_CN was computed using TD-DFT method with the density functional and basis sets aforementioned, and with the Tamm-Dancoff approximation.

### Cell culture and cytotoxicity assay

Human breast cancer cell, MCF-7 and the human retinal pigmented epithelium cell line, RPE, were cultured at 37°C in 5% CO_2_ with Dulbecco's modified Eagle medium (DMEM) and in the presence of 10% fetal bovine serum (FBS) until the confluency reached 50% to 60%. Cell viability was measured by using the PrestoBlue reagent according to the manufacturer's instruction (Invitrogen, USA).

## Author Contributions

W.K.T., L.H.C. and M.M.K.W. carried out all the experiments, W.H.T., H.S.L. and Y.L. performed the data analysis; C.H.L., D.L.M., S.K.C. and C.Y.W. designed the experiments, analyzed the results and wrote the manuscript. * W.K.T. and L.H.C. contributed equally to this work.

## Supplementary Material

Supplementary Information Supplementary Information
