# Supplementary material for: Luminescent Ruthenium(II) Complex Bearing Bipyridine and N-Heterocyclic Carbene-based C∧N∧C Pincer Ligand for Live-Cell Imaging of Endocytosis
Source: Sci Rep. 2015 Mar 13;5:9070. doi: 10.1038/srep09070 (PMC4357851; doi:10.1038/srep09070)
Supplement: Supplementary Information [file srep09070-s1.pdf]

Supporting Information

Luminescent Ruthenium(II) Complex Bearing Bipyridine and N-Heterocyclic Carbene-based C^N^C Pincer Ligand for Live-Cell Imaging of Endocytosis

Wai-Kuen Tsui,[1,¶] Lai-Hon Chung,[1,¶] Matthew Man-Kin Wong,[2] Wai-Him Tsang,[1] Hoi-Shing Lo,[1] Yaxiang Liu,[1] Chung-Hang Leung,[3] Dik-Lung Ma,[4] Sung-Kay Chiu,[*,2] and Chun-Yuen Wong[*,1]

[1]Department of Biology and Chemistry, City University of Hong Kong, Tat Chee Avenue, Kowloon, Hong Kong SAR

[2]Department of Biomedical Sciences, City University of Hong Kong, Tat Chee Avenue, Kowloon, Hong Kong SAR

[3]State Key Laboratory of Quality Research in Chinese Medicine, Institute of Chinese Medical Sciences, University of Macau, Macao, China

[4]Department of Chemistry, Hong Kong Baptist University, 224 Waterloo Road, Kowloon, Hong Kong SAR

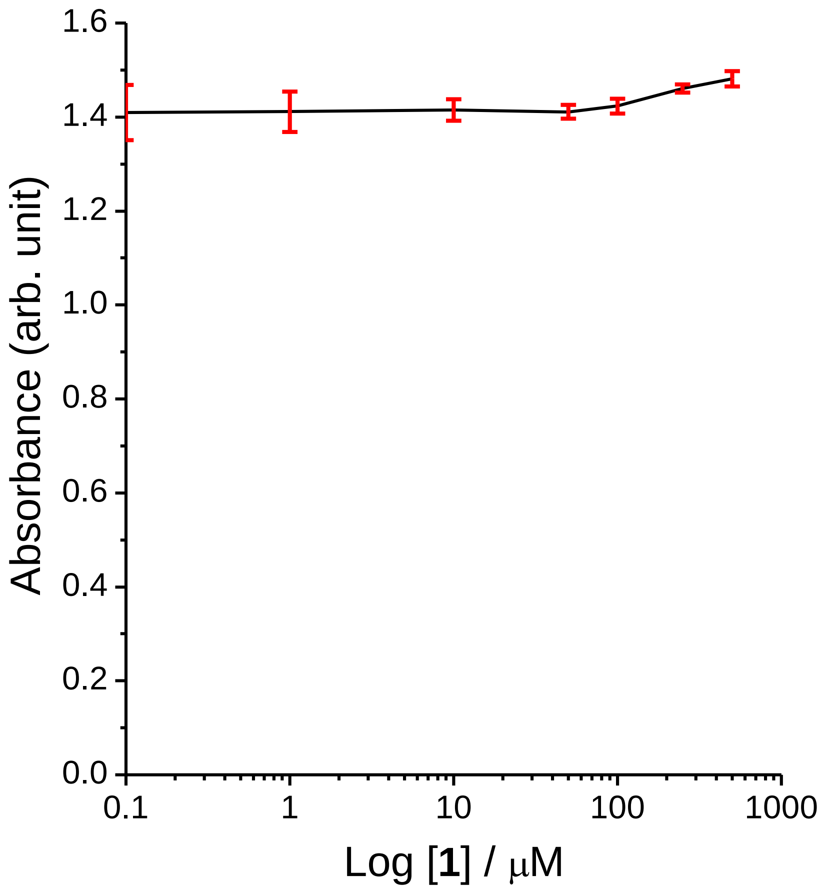

Figure S1
Viability of MCF-7 cells incubated with complex **1**. Cells grown at log-phase were incubated with 0.1 µM, 1 µM, 5 µM, 10 µM, 100µM, 250 µM, and 500 µM for 24h. Cell viability was calculated by the normalization of absorbance at 570 nm to 600 nm using the PrestoBlue assay. The experiments had been triplicated and the standard error bars were plotted.

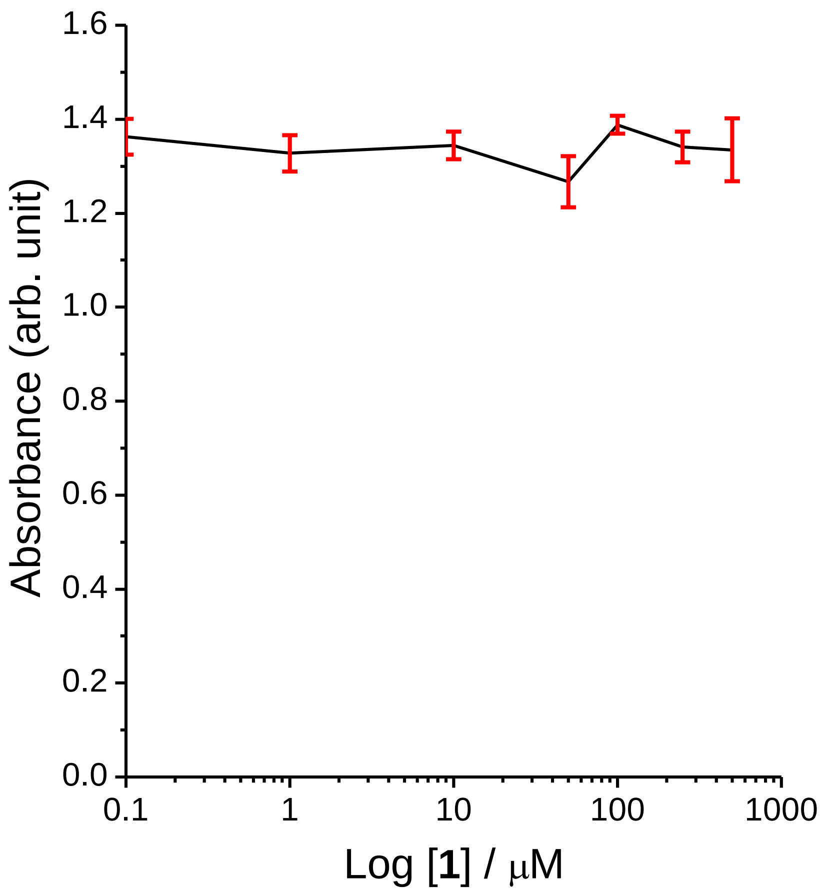

Figure S2
Viability of RPE cells incubated with complex **1**. Cells grown at log-phase were incubated with 0.1 μM, 1 μM, 5 μM, 10 μM, 100μM, 250 μM, and 500 μM for 24h. The experiments had been triplicated and the standard error bars were plotted.

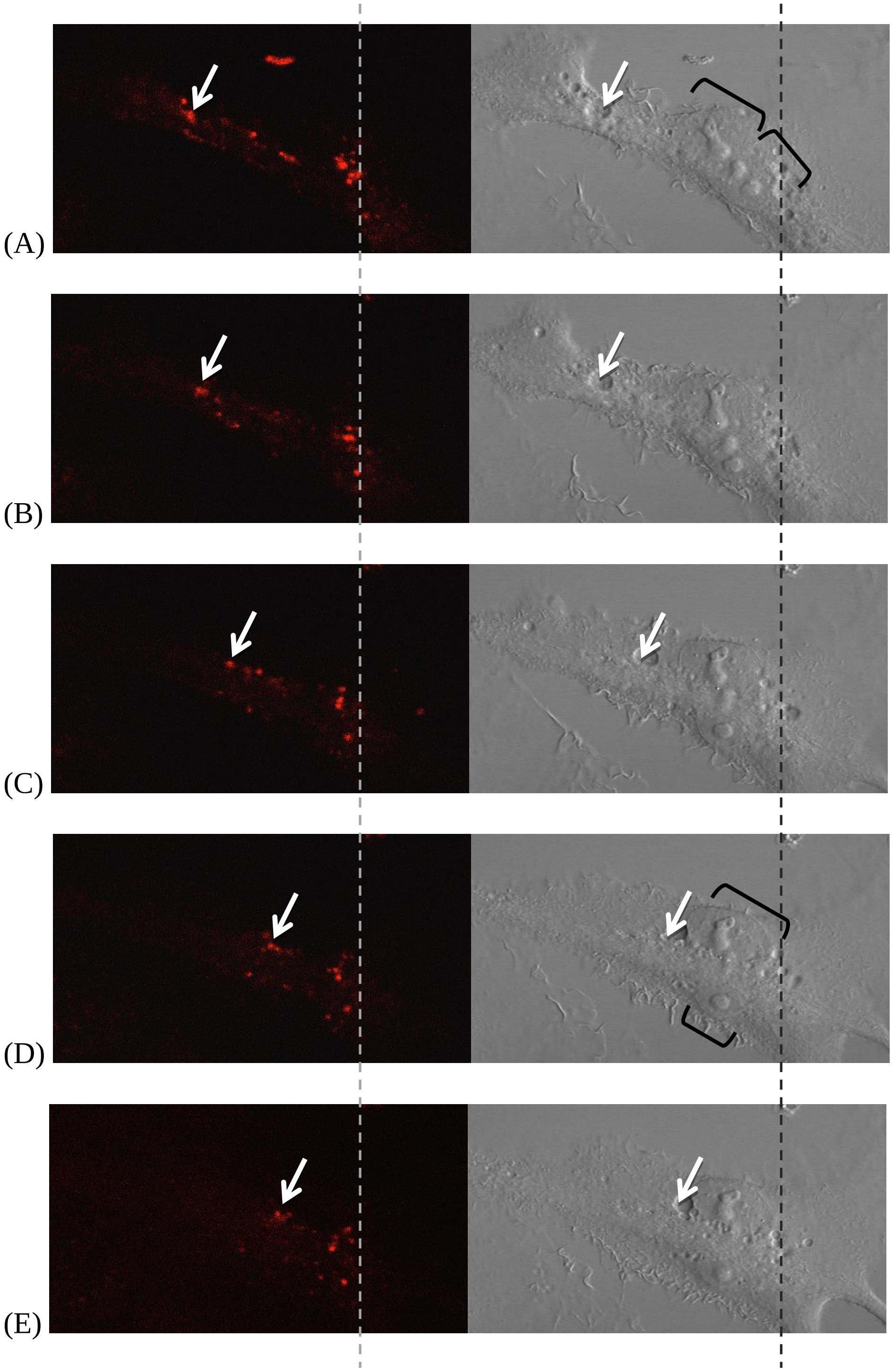

Figure S3
Time-lapse images showing the track of the movement of the luminescent signals from complex **1** in a vesicle (the white arrow) towards the Golgi area of an RPE cell. The relative time frame of the images was: (A) t = 0 min, (B) t= 9 min, (C) t = 18 min, (D) t = 27 min and (E) t = 36 min. Luminescence signals (left column) and DIC images (right column) of the cells were taken at the same time with a confocal microscope. The vertical dotted lines show a fixed position in the optical field to show the movement of the bright vesicles and the nuclei in the cell.
